# Black Cohosh: Insights into its Mechanism(s) of Action

**Published:** 2008-08-27

**Authors:** Rachel L. Ruhlen, Grace Y. Sun, Edward R. Sauter

**Affiliations:** 1 From the Departments of Surgery and; 2 Biochemistry, University of Missouri-Columbia, Columbia, Missouri, U.S.A

**Keywords:** Black cohosh, botanical, complementary and alternative medicine, estrogen, inflammatory, nitric oxide

## Abstract

The Women’s Health Initiative found that combination estrogen and progesterone hormone replacement therapy increases breast cancer and cardiovascular disease risk, which compelled many women to seek herbal alternatives such as black cohosh extract (BCE) to relieve their menopausal symptoms. While several clinical trials document the efficacy of BCE in alleviating menopausal symptoms, preclinical studies to determine how BCE works have yielded conflicting results. Part of this is because there is not a universally accepted method to standardize the dose of black cohosh triterpenes, the presumed active ingredients in the extract. Although the mechanism by which BCE relieves symptoms is unknown, several hypotheses have been proposed: it acts 1) as a selective estrogen receptor modulator, 2) through serotonergic pathways, 3) as an antioxidant, or 4) on inflammatory pathways. We found that while the most prominent triterpene in BCE, 23-epi-26-deoxyactein, suppresses cytokine-induced nitric oxide production in brain microglial cells, the whole BCE extract actually enhanced this pathway. A variety of activities have been reported for black cohosh and its compounds, but the absorption and tissue distribution of these compounds is unknown.

## Introduction

Black cohosh (*Actaea racemosa*, formerly *Cimicifuga racemosa*) is an herb used by menopausal women to alleviate hot flashes and other symptoms of hormone withdrawal. Unlike other herbal medicines used for this purpose, extracts of black cohosh (BCE) have been demonstrated effective for up to three months in most clinical trials. However, the mechanism by which BCE relieves symptoms is unclear. The alleviation of menopausal symptoms by BCE suggests an estrogenic mechanism, but menopausal symptoms can also be alleviated by selective serotonin reuptake inhibitors (SSRIs), suggesting that BCE may work through a serotonergic mechanism. Many menopausal symptoms—hot flashes, mood swings and anxiety, insomnia—are mediated through the central nervous system (CNS) and may be alleviated through a variety of mechanisms. It is possible that BCE can act via multiple tissue-dependent mechanisms, including estrogenic (or antiestrogenic), serotonergic, antioxidative, and inflammatory or antiinflammatory. Herein we review the biological activity of black cohosh, including previously unpublished data supporting a novel mechanism of black cohosh action.

### Standardization of BCE

Like many botanicals, the study of BCE is complicated by the lack of standardization of the extract to one or more active components. Indeed, in the case of BCE it is unclear what the active ingredients are. Moreover, most of the research on efficacy has been conducted on the whole extract and not individual components present in the extract, so it is not known with certainty which components are essential for menopausal symptom relief. Initial reports suggesting estrogenic activity identified formononetin (Jarry et al. 1985), a phytoestrogen found in red clover, as a component of BCE, but later reports failed to find this compound (Struck et al. 1997, Kennelly et al. 2002, Consumer Labs, 2005). Although the active BCE component(s) responsible for alleviation of hot flashes are unknown, a number of triterpenes in BCE have activity individually (Einbond et al. 2004, Burdette et al. 2002, Loser et al. 2000, Kruse et al. 1999, Watanabe et al. 2002), and many BCE preparations are standardized to one or more of the 20 present in BCE (Chen et al. 2002). Routine measurements of all of these compounds are not practical, both due to cost and to the very low levels present for some of the triterpenes. 2-hexylcyclopropaneoctanoic acid has been suggested as a compound unique to BCE and is thus useful for species verification (Panossian et al. 2004). 23-epi-26-deoxyactein (formerly 27-deoxyactein), cimiracemoside A, actein (S) and actein (R) are commercially available for quality control and are the triterpenes most often used for standardization.

The most commonly studied BCEs in clinical trials are Remifemin^®^ and Menofem/Klimadynon^®^. Remifemin^®^ has changed formulation from a 60% ethanol extract to a 40% isopropanol extract, and the extract preparation is not consistently reported in trials (Office of Dietary Supplements, 2002). A Consumer Labs report found that nine black cohosh products and seven combinations of black cohosh with soy or red clover contained expected concentrations of triterpenes (2.5% or label claim) (Consumer Labs, 2005), while a recent study found variability in triterpene glycosides and other constituents among 11 BCE products (Jiang et al. 2006). Variable study outcomes may be due in part to inconsistent triterpene concentrations present in the various preparations used.

### Estrogenic activity of BCE

Many botanicals have been studied for estrogenic activity. Soy, red clover, hops, and other botanicals contain phytoestrogens, the best characterized being the soy isoflavones genistein and daidzein. The use of black cohosh by menopausal women makes the question of estrogenic activity particularly relevant. Table 1 summarizes the findings of whether BCE has estrogenic properties. Similar to estrogen, BCE alleviates hot flashes in humans, reduces depression in a mouse model, and may protect against bone loss in rats and humans (Blumenthal, 2003, Winterhoff et al. 2003, Seidlova-Wuttke et al. 2003b, Wuttke et al. 2003). However, molecular and physiological studies to determine if BCE has estrogen activity have yielded conflicting results. An early study indicated that BCE bound to estrogen receptors (Jarry et al. 1985), while later studies found that it did not (Liu et al. 2001, Onorato and Henion, 2001). The first study used uterine cytosol, and the later studies used recombinant estrogen receptors (ER) α and β. A third study found that BCE competed with estradiol for binding to ERs in uterine cytosol but not to recombinant ERs, confirming the first two seemingly conflicting reports (Jarry et al. 1995). BCE does not induce transcription in estrogen response element reporter assays in yeast (Oerter Klein et al. 2003, Beck et al. 2003), or induce estrogen responsive genes in mammalian endometrial or breast cells, or trout liver cells (Liu et al. 2001, Bennetau-Pelissero et al. 2004). BCE does not induce the proliferation (i.e. not estrogenic) of hormone dependent or independent breast or prostate cancer cell lines, but does inhibit proliferation (antiestrogenic) through the induction of apoptosis (Dixon-Shanies and Shaikh, 1999, Amato et al. 2002, Zierau et al. 2002, Lupu et al. 2003, Bodinet and Freudenstein, 2004, Hostanska et al. 2004a, Jarry et al. 2005, Bodinet and Freudenstein, 2002, Hostanska et al. 2004b, Hostanska et al. 2005). However, like estrogen, BCE induces osteoprotegerin (OPG, a bone marker), progesterone receptor, and ERα in human osteoblast cells, and BCE upregulation of OPG is inhibited by the estrogen receptor antagonist ICI 182,780, implying an estrogenic mechanism (Viereck et al. 2005).

In animals, BCE increases neither uterine weight nor vaginal cell cornification (Einer-Jensen et al. 1996, Liske et al. 2002, Burdette et al. 2003), does not change serum estradiol, testosterone, FSH, prolactin or SHBG (Liske et al. 2002, Zhang et al. 2003), or affect transcription of uterine estrogen responsive genes (Kretzschmar et al. 2005). Similar to estrogen, three day BCE treatment reduced serum LH in ovariectomized rats, although 14 day treatment did not (Jarry and Harnischfeger, 1985). The most consistent evidence of an estrogenic effect of BCE is on the bone (Seidlova-Wuttke et al. 2005, Seidlova-Wuttke et al. 2003a, Seidlova-Wuttke et al. 2003b, Nisslein and Freudenstein, 2003a, Viereck et al. 2005).

Human data are less consistent, and no report has evaluated breast specific estrogenic effects. Early reports suggested an estrogenic effect of BCE (varying preparations and doses) on vaginal cytology and serum hormones. Indices of estrogenic activity in vaginal cells increased after 4 and 12 weeks of BCE (Warnecke, 1985). Twelve weeks of BCE induced vaginal epithelial proliferation (Stoll, 1987). In another study, 12 weeks of BCE did not change endometrial thickness, but slightly increased the number of superficial vaginal cells (Wuttke et al. 2003). In one study, serum LH was reduced by BCE (Duker et al. 1991), while others failed to identify estrogenic effects of BCE in humans, with no effect on circulating LH, FSH, sex hormone binding globulin, estradiol, or prolactin concentrations, or changes in vaginal cytology (Liske et al. 2002) (Lehmann-Willenbrock and Riedel, 1988) (Nappi et al. 2005). These reports of BCE estrogenic activity are consistent with the effects of a selective estrogen receptor modulator (SERM), which acts as an estrogen agonist in some tissues, and as an estrogen antagonist in others. The ideal SERM is one which acts as an estrogen on bone and brain, but does not act as an estrogen in the breast and uterus. BCE may contain compounds which fit the criteria of a SERM.

### Serotonergic activity of BCE

The lack of estrogenic activity in the uterus *in vivo* and breast *in vitro* does not rule out the possibility that BCE alleviates hot flashes by acting as an estrogen in the brain. Alternatively, BCE may alleviate hot flashes by acting on neurotransmitter systems. Selective serotonin reuptake inhibitors (SSRIs) are effective in relieving hot flashes. The SSRIs, while effective in up to 65% of women with hot flashes (Hoda et al. 2003), have side effects which lead many women to stop taking them. While BCE does not inhibit the serotonin transporter, it binds eight serotonin receptor subtypes, acting most strongly on serotonin receptors 5-HT_7_ and 5-HT_1A_ as a mixed competitive ligand, with partial agonist activity on 5-HT_7_ (Burdette et al. 2003). Both 5-HT_7_ and 5-HT_1A_ are found in the hypothalamus and are involved in thermoregulation (Hedlund et al. 2003, Maswood et al. 1995). In serotonergic neurons of the thermoregulatory hypothalamus, 5-HT_1A_ interacts with the serotonin transporter to modulate serotonin reuptake (Lin et al. 1998). Estrogen binding to its receptor interacts with G-protein coupled serotonin receptors, in particular 5-HT_1A_ (Mize et al. 2003). Thus, thermoregulation can be influenced by estrogen, SSRIs, and 5-HT_1A_ and 5-HT_7_ ligands via interactions between receptors and transporters.

### Antioxidant, anti-allergic and anti-inflammatory properties of BCE

Increased oxidative stress and stimulation of inflammatory pathways play an important role in the progression of many neurodegenerative diseases, including Alzheimer’s disease and stroke (Gonzalez-Scarano and Baltuch, 1999). BCE has been used in Korean folk medicine to treat pain and inflammation. A study by Kim et al. (Kim et al. 2004) tested the potential effects of BCE on the allergic response in mast cells. Oral administration of BCE to Sprague-Dawley rats significantly inhibited the anti-IgE-induced passive cutaneous anaphylaxis reaction in a dose-dependant fashion. In addition, BCE inhibited mRNA of cytokines (IL-4, IL-5 and TNF-alpha) induced by the inflammatory agents PMA and A23187 in HMC-1 human leukemia mast cells (Kim et al. 2004).

It is unclear if BCE has antioxidant properties. While a study using the Japanese fish Oryzias latipes did not identify antioxidant effects of BCE and its components (Zhang et al. 2003), another study found that BCE and individual components of BCE had antioxidant properties *in vitro*, suggesting that BCE can protect against DNA damage caused by reactive oxygen species (ROS) (Burdette et al. 2002).

### Biological activity of BCE compounds

**Figure 1.**
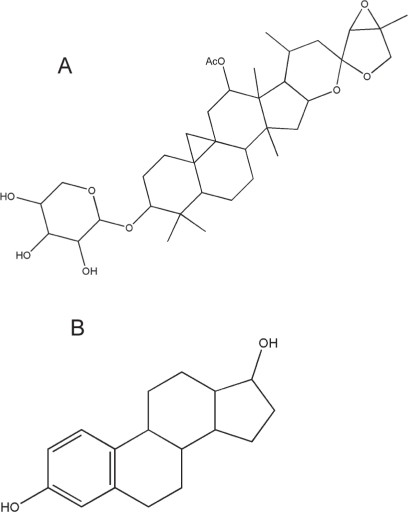
23-epi-26-deoxyactein (A) and 17β-estradiol (B).

Triterpene glycosides and aromatic acids are the main classes of BCE compounds (Chen et al. 2002). Triterpenes, a large and structurally diverse family of chemicals, are found in many plants and in some animals (Xu et al. 2004). Triterpene glycoside conjugates accumulate in plants and form saponins (Jenner et al. 2005). Various triterpenes and saponins have been reported to be anti-cancer, anti-inflammatory, and promote or induce apoptosis (Einbond et al. 2004, Jenner et al. 2005). BCE triterpenes have a five-ring structure similar to the four-ring structure of steroids (Fig. 1). 23-epi-26-deoxyactein is the major triterpene constituent of BCE and is commonly used for standardization (Chen et al. 2002).

Two major BCE fractions containing triterpene glycosides or aromatic acids inhibited MCF-7 cell proliferation and induced apoptosis, with the aromatic acids being more potent (Hostanska et al. 2004b). The triterpene cimiracemoside G was cytotoxic to human oral squamous cell carcinoma but not normal human gingival fibroblasts, suggesting a cancer-specific activity (Watanabe et al. 2002). The triterpenes actein, 23-epi-26-deoxyactein, cimifugoside, and cimiracemoside A inhibited MCF-7 cell proliferation, and actein was shown to induce cell cycle arrest at G1 and inhibit cell cycle proteins cyclin D1 and cdk4 (Einbond et al. 2004). These studies suggest that individual components of BCE may have specific effects on cell metabolism, probably depending on the type and environment of the cells.

Aromatic acids isolated by biofractionation from BCE which offer protection against menadione-induced DNA damage in S30 breast cancer cells included methyl caffeate, caffeic acid, ferulic acid, cimiracemate A, cimiracemate B, and fukinolic acid (Burdette et al. 2002). Aromatic acids caffeic acid, fukinolic acid, and cimicifugic acids A, B, E and F inhibited neutrophil elastase, an enzyme which is elevated in plasma during active inflammation (Loser et al. 2000). While BCE and triterpenes present in BCE inhibit MCF-7 cell proliferation, fukinolic acid increased proliferation (Kruse et al. 1999).

### BCE and neuroinflammation

Microglial cells are CNS macrophages activated by exposure to lipopolysaccharide (LPS), interferon gamma (IFNγ), and other factors (Minghetti and Levi, 1998, Banati et al. 1993). Activated microglia display altered morphology and secrete pro-inflammatory and cytotoxic factors including nitric oxide (NO), prostaglandins, and ROS. Microglial inflammatory factors may protect tissues from infection, but also induce neurode-generation.

While the constitutive production of low concentrations of NO by endothelial nitric oxide synthase (eNOS) or neuronal NOS (nNOS) is necessary to maintain physiologic functions, excess production of NO (100–1000 times more) by inducible NOS (iNOS) is harmful (Kuo and Abe, 1995). NO reacts with superoxide to form peroxynitrite anions, which in turn decompose to hydroxyl free radicals and nitrogen dioxide (Beckman et al. 1990). These compounds can target proteins and nucleic acid and inhibit function by forming nitrosyl derivatives. Excess NO production has been linked with cell death in neurodegenerative diseases and other brain injuries (Dawson and Snyder, 1994, Moncada and Higgs, 1993).

The majority of mechanistic information about black cohosh concerns whether or not it is estrogenic. Because hot flashes originate in the CNS, black cohosh effects may bypass or indirectly interact with estrogenic systems. It has been suggested that estrogen effects on the CNS involve the NO pathway (Lopez-Jaramillo and Teran, 1999), and NO controls release of anterior pituitary hormones (McCann et al. 1998). The activation of iNOS in stress-induced temperature increase (Soszynski, 2006) suggests a possible non-estrogenic thermoregulatory mechanism for black cohosh. In this study, we examined the effect of black cohosh and two of its triterpene glycosides, 23-epi-26-deoxyactein and cimiracemoside A, on the induction of NO in murine BV-2 microglial cells by the bacteria endotoxin LPS and by IFNγ. Our previous studies demonstrated that LPS and IFNγ can induce iNOS through different signaling pathways in these cells (Shen et al. 2005).

## Methods

### Materials

The BCE preparation CimiPure^®^ containing 2.5% triterpene glycosides was obtained from PureWorld (South Hackensack, NJ), while 23-epi-26-deoxyactein was purchased from Chromadex (Santa Ana, CA). Black cohosh roots and rhizomes from the University of Missouri Botanical Center were powdered. All test chemicals were dissolved in dimethylsulfoxide prior to use.

### Cells

Immortalized BV-2 murine microglia cells were maintained in DMEM with 5% FBS, 1% penicillin/streptomycin and 1% fungizone. RAW 264.7 murine macrophage cells were maintained in DMEM with 10% FBS, 1% penicillin/streptomycin and 1% fungizone. The cells were grown to 90% confluence in 12- or 24- well plates. BV-2 cells were serum starved for 4 hours before overnight treatment with IFNγ (R&D Systems, Minneapolis, MN or Chemicon, Temecula, CA) or LPS (Sigma, St. Louis, MO). RAW 264.7 cells were treated overnight with IFNγ (R&D Systems, Minneapolis, MN or Chemicon, Temecula, CA) or LPS, and were not serum starved. We conducted a dose-response to determine the optimal concentrations of cytokines to induce at least 20 μM NO production, which is approximately 50% of maximal stimulation. Cells were pretreated for 30 minutes with BCE or 23-epi-26-deoxyactein prior to exposure to IFNγ or LPS. NO was determined by measuring levels of nitrite in the medium using the Greiss reaction.

### RT-PCR

RNA was isolated using an RNEasy Mini Kit (Qiagen, Valencia, CA). Total RNA was quantified by spectrophotometer. Primer sequences were 5′-GACAAGCTGCATGTGACATC-3′ and 5′-GCTGGTAGGTTCCTGTTGTT-3′ for iNOS and 5′-TGGAGAAGAGCTATGAGCTGCCTG-3′ and 5′-GTGCCACCAGACAGCACTGTGTTG-3′ for β-actin. Superscript III One Step (Invitrogen, Carlsbad, CA) was used for RT-PCR with 16 μg total RNA. iNOS was amplified 25 cycles and β-actin for 30 cycles.

### Statistics

Data were analyzed by ANOVA considering the effect of treatment in the model using the Statistical Analysis System (SAS). If the main effect was significant, least squared difference was used for means separation. A *P*-value of less than 0.05 was considered statistically significant. All data are expressed as mean ±SE from at least three experiments.

## Results

**Figure 2.**
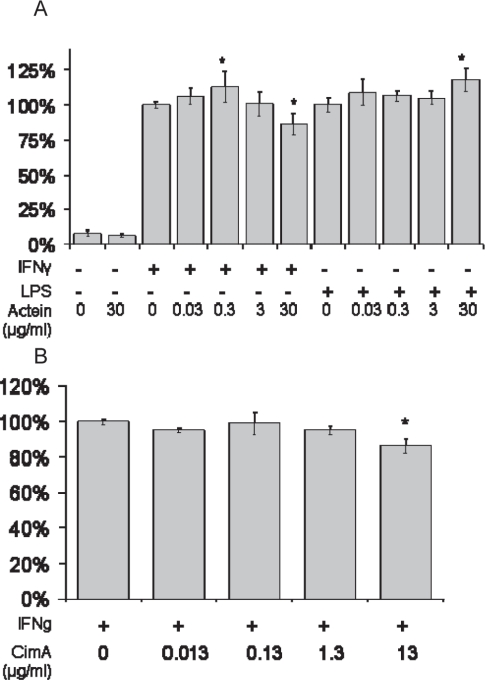
NO production from cytokine-induced BV-2 cells pretreated with A) 23-epi-26-deoxyactein (Actein) or B) cimiracemoside-A (CimA). Data are expressed as mean % of cytokine-treated control ± SE. *P < 0.05 vs cytokine control.

Typically, unstimulated BV-2 cells produced less than 5 μM NO. BV-2 cells stimulated for 18 hours with IFNγ produced 38 ± 7 μM NO. BV-2 cells stimulated for 18 hours with LPS produced 29 ± 6 μM NO. 30 μg/ml 23-epi-26-deoxyactein had no effect on NO production, but inhibited IFNγ-induced NO production in a dose dependent fashion up to 14%, with a slight increase observed at the lower doses (0.3 μg/ml) of 23-epi-26 deoxyactein (Fig. 2A). 30 μg/ml 23-epi-26-deoxyactein slightly stimulated LPS-induced NO production (Fig. 2A). 13 μg/ml cimiracemoside A slightly decreased NO production after induction by IFNγ (Fig. 2B).

**Figure 3.**
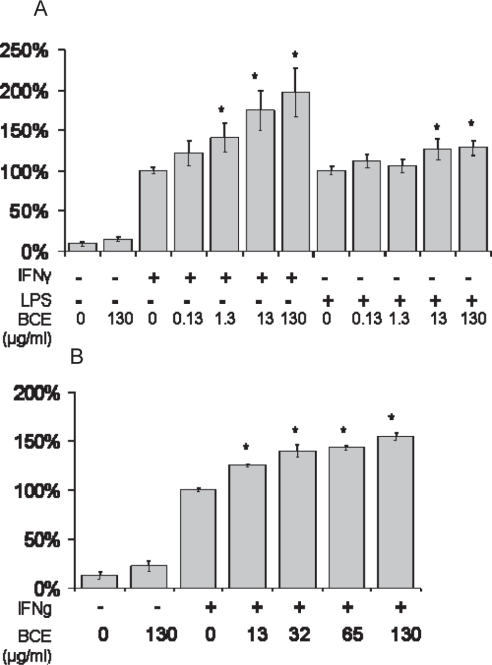
NO production from cytokine-induced BV-2 cells pretreated with black cohosh from PureWorld (A), or MU Botanical Center (B). Data are expressed as mean % of cytokine-treated control ± SE. *P < 0.05 vs cytokine control.

Addition of 130 μg/ml BCE did not alter NO production in BV-2 cells, but increased NO production in IFNγ-stimulated cells in a dose-dependent fashion from 0.13 to 130 μg/ml BCE. Specifically, addition of 130 μg/ml BCE increased NO production by 97 ± 30% over IFNγ alone (Fig. 3A). BCE (13 and 130 μg/ml) slightly increased NO production in LPS-stimulated cells. To determine if this effect was consistent across different sources of black cohosh, black cohosh harvested from the MU Botanical Center was obtained. This second BCE source (13 to 130 μg/ml) also induced NO production in a dose dependent fashion, with 54 ± 4% increase at 130 μg/ml over IFNγ alone (Fig. 3B).

**Figure 4.**
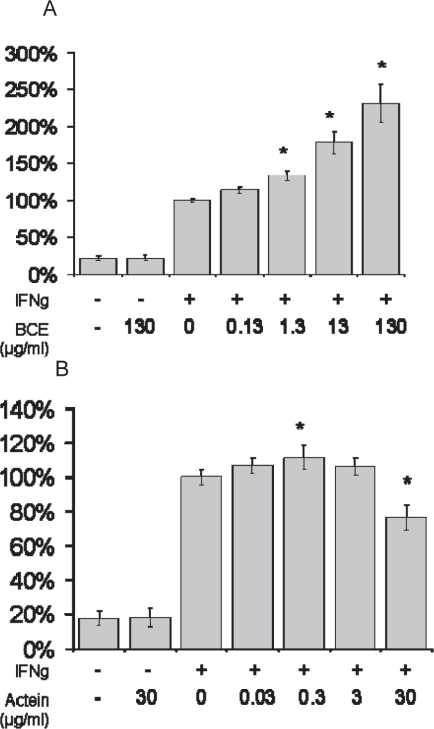
NO production from cytokine-induced RAW cells pretreated with A) black cohosh (PureWorld) or B) 23-epi-26-deoxyactein. Data are expressed as mean % of cytokine-treated control ± SE. *P < 0.05 vs cytokine control.

The effects of BCE and 23-epi-26-deoxyactein on NO production were also tested in RAW 264.7 cells. BCE stimulated IFNγ-induced NO production an additional 131 ± 26% over IFNγ alone in a dose dependent fashion from 0.13 to 130 μg/ml, while 30 μg/ml 23-epi-26-deoxyactein suppressed NO production by up to 23 ± 7%, with a slight increase observed at the lower doses of 0.3 μg/ml (Fig. 4).

**Figure 5.**
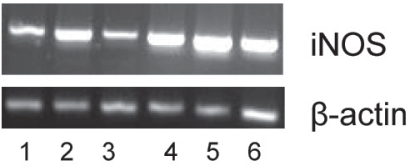
iNOS mRNA from IFNγ-induced BV-2 cells pretreated with black cohosh or 23-epi-26-deoxyactein. Lane 1, no treatment. Lane 2, 130 μg/ml BCE. Lane 3, 30 μg/ml 23-epi-26-deoxyactein. Lane 4, IFNγ. Lane 5, IFNγ + 130 μg/ml BCE. Lane 6, IFNγ + 30 μg/ml 23-epi-26-deoxyactein. Results were repeated 3 times in triplicate, representative RT-PCR shown.

iNOS mRNA in BV2 cells was increased relative to β-actin control by 130 μg/ml BCE or IFNγ, and a further increase was observed with BCE and IFNγ combined (Fig. 5). 23-epi-26-deoxyactein (30 μg/ml) did not have an observable effect on iNOS mRNA in IFNγ-stimulated or unstimulated cells.

## Discussion

We identified, for the first time, biological activity of both the whole extract and the most prominent triterpene in BCE, 23-epi-26-deoxyactein, in conjunction with the induction of NO production in microglial cells and macrophages. In general, neither BCE nor 23-epi-26-deoxyactein exerted effects on LPS-induced NO production in these cells. However, while 23-epi-26-deoxyactein decreased IFNγ-induced iNOS mRNA and NO production, BCE had the opposite effect. This effect was consistent in both BV-2 microglia and RAW 264.7 macrophages. The lack of effect of BCE on the LPS pathway is in line with the notion that this product would not be effective in the treatment of inflammatory diseases. The opposing effect of BCE and 23-epi-26-deoxyactein on IFNγ-induced NO production suggests that components present in BCE other than 23-epi-26-deoxyactein may be responsible for the elicited effects. Synthetic triterpenes tested in a similar system had the same effect as 23-epi-26-deoxyactein, suppressing iNOS expression and NO production (Suh et al. 1998). The specificity of these compounds on the IFNγ pathway is intriguing since immune cell IFNγ is an important innate factor for the body’s immune defense system (Owens et al. 2005). Thus, the ability of BCE to enhance NO production under suboptimal levels of IFNγ may represent a new mechanism of action of BCE in modulating the immune defense system. Obviously, more studies are needed to explore the physiological significance of the observations resulting from this study.

While constitutive NOS (eNOS and nNOS) production is regulated post-transcriptionally, iNOS synthesis is primarily regulated transcriptionally (Nathan and Xie, 1994). BCE alone was able to slightly upregulate iNOS activity, but its effects were most pronounced in conjunction with suboptimal levels of IFNγ. Inflammatory systems may play a role in the mediation of hot flashes. It is unknown whether the pro-inflammatory effects of BCE or the anti-inflammatory effects of select BCE-compounds such as 23-epi-26-deoxyactein impact thermoregulation and thus hot flashes.

It is clear that BCE acts through a variety of biological pathways. Our evidence supports mechanisms involving inflammatory, rather than estrogenic, pathways. The role of specific chemicals or classes of chemicals, i.e. triterpenes, should be examined in these systems. To determine physiological relevance it will be necessary to understand the pharmacokinetics of BCE: how it is absorbed, metabolized, tissue distribution, and clearance. In particular, since many of the post-menopausal symptoms are thought to arise from the CNS, more studies are needed to find out if specific BCE components can cross the blood-brain barrier and exert effects to regulate the CNS thermoregulation system.

### Hepatotoxicity

There have been a handful of case reports linking black cohosh to liver damage (Levitsky et al. 2005, Cohen et al. 2004, Lontos et al. 2003, Whiting et al. 2002), (reviews in (Thomsen et al. 2004, Huntley, 2004, Huntley and Ernst, 2003)). Conclusions from these case reports were confounded by the lack of details reported, for example, dose, source, and preparation of black cohosh. For example, in the case in which a liver transplant was necessary, the woman had been taking 500 mg black cohosh extract daily, whereas the generally recommended daily dose is 20 or 40 mg (Levitsky et al. 2005). In another case report, hepatitis symptoms continued four months after discontinuation of black cohosh, so the hepatitis is unlikely related to the herb (Cohen et al. 2004). Herbs other than black cohosh were being ingested by subjects in some case reports, including ground ivy which contains pugelone, a known liver toxicant (Thomsen et al. 2004). Using WHO criteria, in which cases are categorized as unclassifiable, unclassified, unlikely, possible, probably, or certain, to evaluate the link between hepatic toxicity and black cohosh, of three case reports reviewed, two cases were deemed unclassified and one case possible (Huntley and Ernst, 2003). Two clinical trials evaluating the efficacy of black cohosh which measured liver enzymes before and after treatment found no evidence of change in enzyme levels (Osmers et al. 2005, Nappi et al. 2005).

In summary, the literature to date does not support a direct estrogenic mechanism of BCE to explain its effects, but it may act through systems involving neurotransmitters and inflammatory pathways. The efficacy of BCE is supported by many, but not all, clinical trials, and there is little evidence of toxicity or severe adverse effects due to BCE. Women and clinicians want to know if BCE works, how it works, and if it is safe. These questions cannot yet be answered with absolute confidence, but the evidence supports its efficacy, and to date there is little evidence of toxicity, at least short-term.

## Figures and Tables

**Table 1.** Estrogenic activity of black cohosh.

	**Estrogenic**	**Not estrogenic**	**Antiestrogenic**
Serum hormones	2 studies^1^, Single dose BCE^2^	3 studies^3^, ^4^, ^5^	
Uterine or vaginal tissue		7 studies^2^, ^3^, ^6^, ^7^, ^8^, ^9^, ^10^	
ER binding (cytosol)	3 studies^1^, ^2^, ^11^, ^12^		
ER binding (recombinant)		3 studies^12^, ^13^, ^14^	
ERE-reporter		5 studies^7^, ^15^, ^16^, ^17^, ^18^	High dose^15^
Stimulate ER responsive genes	High dose^19^	7 studies^2^, ^3^, ^5^, ^10^, ^13^, ^17^, ^20^, ^21^	
*In vivo* tumor growth		2 studies^3^, ^22^	1 study^23^
*In vitro* cancer cell growth	BCE compound fukinolic acid^24^	2 studies^7^, ^17^	7 studies^15^, ^25^, ^26^, ^27^, ^28^, ^29^, ^30^
Bone markers	4 studies^2^,^9^, ^20^, antagonized by ICI 182780^21^	1 study^31^	
Bone density	3 studies^2^, ^23^, ^31^		

In general, estrogens induce a decrease in LH or FSH, increase uterine weight and vaginal cytology, compete estradiol binding to uterine cytosol or recombinant ER, stimulate ERE-reporter assays, ER responsive genes, promote estrogen responsive tumor growth *in vivo* and *in vitro*, increase or decrease bone formation or resorptive markers, and increase bone density.

1 (Duker et al. 1991),

2 (Seidlova-Wuttke et al. 2003a),

3 (Freudenstein et al. 2002),

4 (Liske et al. 2002),

5 (Zhang et al. 2003),

6 (Einer-Jensen et al. 1996),

7 (Amato et al. 2002),

8 (Burdette et al. 2003, Burdette et al. 2002),

9 (Wuttke et al. 2003),

10 (Kretzschmar et al. 2005),

11 (Jarry et al. 1985),

12 (Jarry et al. 1995),

13 (Liu et al. 2001),

14 (Onorato and Henion, 2001),

15 (Zierau et al. 2002),

16 (Beck et al. 2003),

17 (Lupu et al. 2003),

18 (Oerter Klein et al. 2003),

19 (Wober et al. 2003),

20 (Bennetau-Pelissero et al. 2004),

21 (Viereck et al. 2005),

22 (Nisslein and Freudenstein, 2004),

23 (Nisslein and Freudenstein, 2003b),

24 (Kruse et al. 1999),

25 (Dixon-Shanies and Shaikh, 1999),

26 (Bodinet and Freudenstein, 2002),

27 (Bodinet and Freudenstein, 2004),

28 (Hostanska et al. 2004b),

29 (Hostanska et al. 2004a),

30 (Jarry et al. 2005),

31 (Seidlova-Wuttke et al. 2005).

## Abbreviations:

BCE: black cohosh extract
SSRI: selective serotonin reuptake inhibitor
CNS: central nervous system
ER: estrogen receptor
OPG: osteoprotegerin
SERM: selective estrogen receptor modulator
ROS: reactive oxygen species
LPS: lipopolysaccharide
IFNγ: interferon γ
NO: nitric oxide
NOS: nitric oxide synthase
